# Molecular mechanisms mediating root hydrotropism: what we have observed since the rediscovery of hydrotropism

**DOI:** 10.1007/s10265-019-01153-3

**Published:** 2019-12-04

**Authors:** Yutaka Miyazawa, Hideyuki Takahashi

**Affiliations:** 1 Faculty of Science, Yamagata University, 1-4-12 Kojirakawa-machi, Yamagata, 990-8560 Japan; 2 Graduate School of Life Sciences, Tohoku University, 2-1-1 Katahira, Aoba-ku, Sendai, 980-8577 Japan

**Keywords:** Abscisic acid, Auxin, Hydrotropism, MIZU-KUSSEI1 (MIZ1), MIZU-KUSSEI2 (MIZ2)

## Abstract

Roots display directional growth toward moisture in response to a water potential gradient. Root hydrotropism is thought to facilitate plant adaptation to continuously changing water availability. Hydrotropism has not been as extensively studied as gravitropism. However, comparisons of hydrotropic and gravitropic responses identified mechanisms that are unique to hydrotropism. Regulatory mechanisms underlying the hydrotropic response appear to differ among different species. We recently performed molecular and genetic analyses of root hydrotropism in *Arabidopsis thaliana*. In this review, we summarize the current knowledge of specific mechanisms mediating root hydrotropism in several plant species.

## Introduction

Plants have evolved innovative mechanisms to respond to environmental cues, acquire limited resources, and adapt to biotic and abiotic stresses. Tropic responses involve directional growth of a plant organ or the whole plant toward or away from environmental stimuli including light, gravity, touch, salt, moisture gradient, and magnetic field (Fasano et al. 2002; Galvan-Ampudia et al. 2013; Jaffe et al. 1985; Porterfield and Musgrave 1998; Vandenbrink and Kiss 2019; Whippo and Hangarter 2006). Shoots exhibit positive phototropism or negative gravitropism to sunlight, whereas roots exhibit negative phototropism or positive gravitropism to avoid desiccation and anchor into the soil. Roots also exhibit positive hydrotropic growth in response to moisture gradients, which has long been considered to contribute to drought avoidance (Knight 1811).

**Fig. 1:**
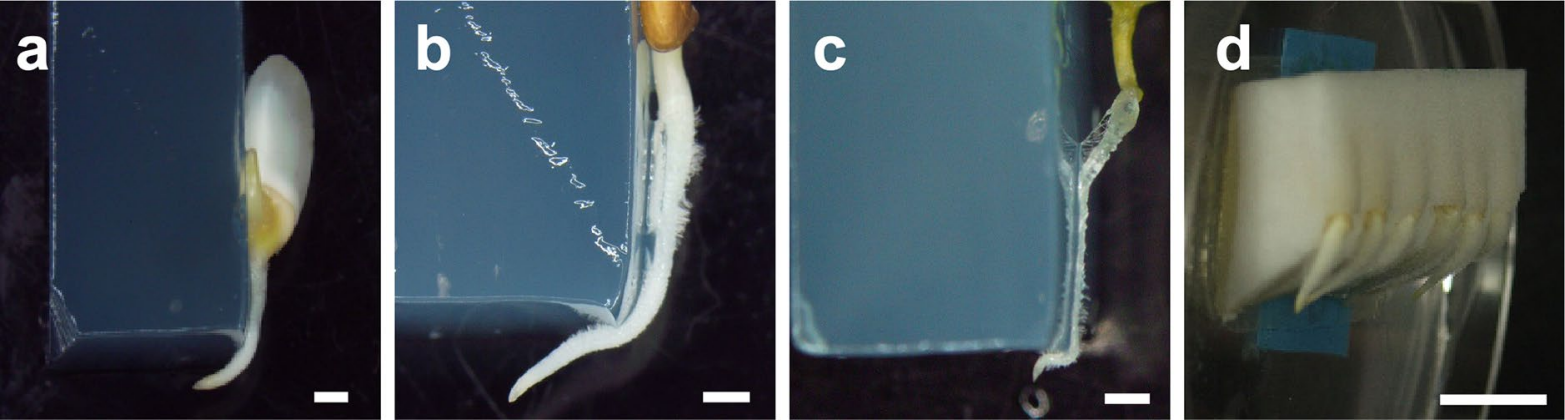
Hydrotropic responses of various plant species. Seedling roots of rice (*Oryza sativa*) (**a**), *Lotus japonicus* (**b**), tobacco (*Nicotiana tabacum*) (**c**), and cucumber (*Cucumis sativus*) (**d**) were hydrostimulated for 12 h and then photographed. Scale bars in **a**, **b**, and **c** indicate 1 mm; scale bar in **d** indicates 1 cm

**Fig. 2:**
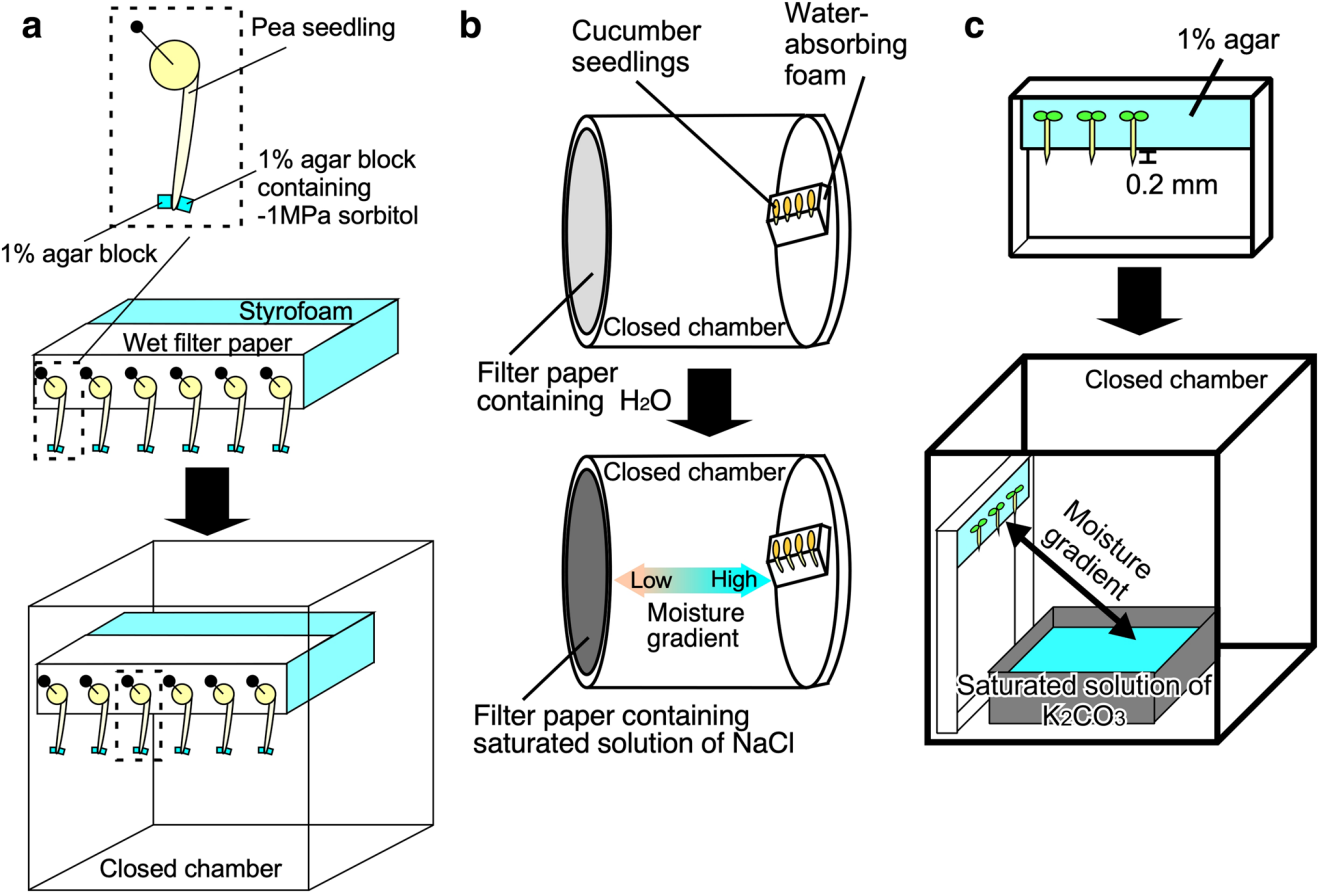
Experimental systems for studying root hydrotropism. Experimental set ups for inducing hydrotropic responses in pea (*Pisum sativum*) (**a**), cucumber (**b**) and Arabidopsis (*Arabidopsis thaliana*) (**c**) are shown. **a** Agar blocks either containing -1MPa sorbitol or not were applied to both sides of the root tip end. The seedlings were fixed to the Styrofoam covered with moistened filter paper. Then, they were enclosed into a plastic chamber. **b** Cucumber seeds inserted into the pockets made on the water-absorbable foam were germinated under 1G condition without moisture gradient. After germination, filter paper containing water was replaced with a filter paper containing saturated sodium chloride solution to establish a moisture gradient across the plastic foam and the filter paper. Just after the replacement of the filter paper, seedlings were grown under clinorotating condition. **c** Arabidopsis seedlings were aligned on 1% agar plates with their root tip suspended freely into the air. Then the agar plate was enclosed in a plastic chamber in which a moisture gradient is established between 1% agar and a saturated solution of potassium carbonate set on the base of the chamber. Note that hydrotropic responses of *Lotus japonicus* and rice seedling roots were induced using this system

Phototropism and gravitropism have been studied extensively, whereas there are fewer studies on the mechanisms mediating root hydrotropism. This is partly due to technical challenges in designing experiments that eliminate gravitropic effects, and the fact that root hydrotropism is often dominated by gravitropism (reviewed in Takahashi 1997). Jaffe et al. (1985) analyzed the roots of an agravitropic pea (*Pisum sativum* L.) mutant, *ageotropum*, which displayed distinct bending toward a moisture gradient and no bending when the moisture gradient was absent. This experiment established root hydrotropism as a genuine response to a moisture gradient, and indicated that root gravitropism interfered with the expression of root hydrotropism, at least in wild-type pea. In addition, a spaceflight experiment demonstrated that lateral roots of cucumber (*Cucumis sativus* L.) seedlings exhibited obvious hydrotropic growth under microgravity condition (Takahashi et al. 1999). Since the roots of cucumber do not display hydrotropism under 1G condition, it was shown that gravitropism interfered with hydrotropism also in cucumber. These data led to the development of new experimental systems for observing root hydrotropic responses in several species (Figs. 1, 2a, b), including wheat (*Triticum aestivum* L.), maize (*Zea mays* L.), and cucumber (Mizuno et al. 2002; Oyanagi et al. 1995; Takahashi and Scott 1991). These studies primarily focused on the physiological features of hydrotropism in comparison with those of gravitropism. The results of these studies showed that (1) gravitropism interferes with hydrotropism, (2) water potential gradients are perceived in the root cap, and (3) Ca^2+^ and auxin are involved in hydrotropic responses (reviewed in Takahashi et al. 2009).

Experimental systems to study the molecular mechanisms mediating root hydrotropism in *Arabidopsis thaliana* were established in 2002 (Fig. 2c; Takahashi et al. 2002). Eapen et al. (2003) also developed a system for inducing the hydrotropic response in Arabidopsis, and isolated the first ahydrotopic mutant *no hydrotropic response1 (nhr1)*. A subsequent forward-genetics approach isolated two ahydrotropic mutants, *mizu-kussei1* (*miz1*) and *miz2*, along with their responsible genes *MIZ1* and *MIZ2*, respectively (Kobayashi et al. 2007; Miyazawa et al. 2009). These breakthroughs stimulated research on the molecular mechanisms mediating hydrotropism and analyses of how these differ across species. The data consistently indicate that plants express both common and species-specific mechanisms to mediate root hydrotropism (Table 1.). This review summarizes the current knowledge obtained from molecular biological, and comparative studies of root hydrotropism, and discusses future research directions.

**Table 1:** Species-specific difference of the mechanism underlying root hydrotropism

Species (family)	Site of stimulus perception	Intracellular signaling mechanism	Intercellular signaling mechanism	Growth mechanism	References
Arabidopsis (Brassicaceae)	Root cap, elongation zone	Increment of cytosolic Ca^2+^	Ca^2+^-mediated signaling	ABA-dependent differential growth of cortical cells	Dietrich et al. (2017), Shkolnik et al. (2018), Takahashi et al. (2003)
Cucumber (Cucurbitaceae)	Root cap, elongation zone	N.D.	Auxin polar transport	Auxin-dependent differential growth of epidermal cells	Fujii et al. (2018,) Morohashi et al. (2017)
*Lotus japonicus* (Fabaceae)	Root cap, elongation zone	Auxin synthesis	N.D.	Auxin response independent of TIR/AFB pathway	Nakajima et al. (2017)
Pea (Fabaceae)	Root cap	Increment of cytosolic Ca^2+^	Auxin polar transport	Auxin-dependent differential growth of epidermal cells	Jaffe et al. (1985), Nakajima et al. (2017), Takano et al. (1995)
Rice (Poaceae)	Root cap, elongation zone	N.D.	Auxin polar transport	Auxin-dependent differential growth of epidermal cells	Nakajima et al. (2017)

N.D., not determined

### The Cholodny-Went theory accurately explains root hydrotropism for some plant species

There is a consensus that stimulus-dependent auxin redistribution along a plant organ causes a differential growth response that results in the organ bending, which is referred to as Cholodny-Went theory (Went and Thimann 1937). Emerging evidence suggested that shoot and root gravitropic responses and shoot phototropic response also are explained by the Cholodny-Went theory (reviewed in Horm et al. 2013; Muday 2001; Nakamura et al. 2019). After the first experimental breakthrough in root hydrotropism (Jaffe et al. 1985), researchers evaluated similarities between hydrotropic response mechanisms and the mechanisms mediating other tropisms. An early study reported that hydrotropism and gravitropism of pea roots were both inhibited by 2,3,5-triiodobenzoic acid (TIBA), which effectively inhibits auxin efflux (Takahashi and Suge 1991). This involvement of auxin transport was confirmed using other auxin efflux inhibitors, including 9-hydroxyfluorene-9-carboxylic acid (HFCA) and 1-naphthylphtalamic acid (NPA) (Nakajima et al. 2017). Nakajima et al. (2017) also demonstrated that pea root hydrotropism was reduced by auxin influx and activity inhibitors. These combined results indicate that hydrotropic bending in pea roots is explained by the Cholodny-Went theory. Similarly, the hydrotropic response in cucumber seedling roots is blocked by TIBA and HFCA and is attenuated by *p*-chlorophenoxy isobutyric acid (PCIB) (Morohashi et al. 2017).

As described above, root hydrotropic response of cucumber is masked by gravitropism under 1G conditions on Earth. To elucidate the molecular mechanism underlying root hydrotropism in such plant species, it is necessary to differentiate hydrotropism from gravitropism. To achieve this, cucumber seedling roots grown under µG in space, together with clinorotated samples, were subjected to molecular biological analyses. Thirteen out of 23 *Aux/IAA* genes displayed asymmetrical expression profiles in hydrotropically responding cucumber roots (Fujii et al. 2018; Mizuno et al. 2002; Morohashi et al. 2017). Some *CsIAA* genes displayed asymmetrical expression profiles in gravitropically bending cucumber roots (Fujii et al. 2018). CsPIN5, a functional homolog of Arabidopsis AtPIN2, displayed asymmetrical expression in cucumber seedling roots subjected to clinorotation and micrgravity (µG) conditions, with higher expression levels in the humid (concave) side than the dry (convex) side of hydrotropically responding roots (Morohashi et al. 2017). Asymmetrical localization of CsPIN5 was also observed in gravitropically responding cucumber roots, with higher expression levels in the lower (concave) side than the upper (convex) side (Morohashi et al. 2017). The Arabidopsis AtPIN2 protein also displayed asymmetrical distribution profiles during the gravitropic response (Abas et al. 2006). These combined results indicate that hydrotropic bending in cucumber roots is explained by the Cholodny-Went theory.

The roles of root cap cells in hydrotropic responses differ between pea and cucumber. In pea, de-tipping the root tip where gravisensing columella cells reside is sufficient to abolish root hydrotropism (Jaffe et al. 1985). Unilateral application of high sorbitol concentrations to the root cap was sufficient to induce the root hydrotropic response in pea (Takano et al. 1995). De-capped maize roots also failed to develop hydrotropic curvature (Takahashi and Scott 1993). These results suggest that the mechanisms for sensing/responding to water potential gradients reside in root cap cells. By contrast, de-tipped cucumber roots displayed significant hydrotropic bending, even under stationary 1 G conditions (Fujii et al. 2018). Asymmetrical expression profiles of auxin-inducible genes were measured in hydrotropically responding de-tipped cucumber roots, and resembled the levels observed in hydrotropically bending intact cucumber roots (Fujii et al. 2018).

Our recent analyses of mechanisms mediating the hydrotropic response in rice (*Oryza sativa* L.) seedlings indicated that they resembled those observed in cucumber seedlings, although rice seedling roots displayed a distinct hydrotropic response under stationary (1 G) conditions (Nakajima et al. 2017). Auxin transport inhibitors disrupted gravitropism and hydrotropism in rice seedling roots but did not reduce root growth (Nakajima et al. 2017). Similar reductions in gravitropic and hydrotropic rice seedling root bending were observed after treatment with auxin synthesis and response inhibitors. These combined results indicate that hydrotropic bending in rice roots is explained by the Cholodny-Went theory. De-tipping the root cap of rice seedlings abolished the gravitropic response, whereas it did not affect the hydrotropic response (Nakajima et al. 2017). This result suggests that there are likely other mechanisms that induce differential auxin distribution without root cap cells. These results highlight the mechanistic difference of root hydrotropism between pea, cucumber and rice.

### The Cholodny-Went theory does not explain root hydrotropism for other plant species

Arabidopsis seedling roots were much more sensitive to moisture gradients and exhibited distinct hydrotropic curvature under stationary conditions compared to pea and cucumber (Takahashi et al. 2002). These features of Arabidopsis roots were ideal for physiological and genetic screening studies because no special equipment was required to nullify gravitropic effects. Owing to this feature, several groups began to analyze hydrotropic response genetically. Arabidopsis mutants reported to exhibit abnormal hydrotropism so far are listed in Table 2. At first, we examined hydrotropic responses in the following Arabidopsis agravitropic mutants: *axr1*-*3* and *axr2*-*1* (auxin response mutants), *aux1*-*7* and *wav6*-*52* (auxin transport mutants), and *pgm* (starchless mutant). Unexpectedly, the hydrotropic responses were slightly enhanced in these five mutants (Takahashi et al. 2002, 2003), suggesting that hydrotropic mechanisms in Arabidopsis roots differ from those in pea, cucumber, and rice. The Arabidopsis root hydrotropic response also was not affected by auxin transport inhibitors (Kaneyasu et al. 2007), and asymmetrical auxin redistribution was not observed during the Arabidopsis root hydrotropic response (Shkolnik et al. 2016; Takahashi et al. 2009). However, it is not clear whether auxin response is required for Arabidopsis hydrotropic response. Treating Arabidopsis seedlings with PCIB substantially reduced hydrotropic bending, whereas treatment with 2-(1H-indol-3-yl)-4-oxo-4-phenylbutanoic acid (PEO-IAA, an auxin antagonist) or auxinole (a specific inhibitor of TIR1/AFB auxin receptors) enhanced hydrotropic bending (Kaneyasu et al. 2007; Shkolnik et al. 2016). Agravitropic auxin response mutants did not display reduced hydrotropic response (Takahashi et al. 2002), suggesting that Arabidopsis root auxin responses are more important for gravitropism than for hydrotropism. Kimura et al. (2018) reported that asymmetrical auxin redistribution is not required for phototropic root bending in Arabidopsis. Although auxin efflux mutants and wild-type plants treated with PEO-IAA displayed enhanced phototropic root bending, some auxin response mutants (*arf7arf19*, *axr3*-*3*, and *tir1*-*1*) displayed slight reductions in the phototropic response (Kimura et al. 2018). The *miz1*-*1* and *miz2* mutants with defective hydrotropic responses displayed reduced phototropic responses (Kobayashi et al. 2007; Takahashi et al. 2009). These results suggest that hydrotropic and phototropic bending mechanisms share a common pathway, at least to some extent. Thus, specific auxin response components may have a role in the hydrotropic response. Future studies should clarify these relationships.

**Table 2:** List of *Arabidopsis thaliana* mutants which exhibit abnormal root hydrotropism

Mutant	Biological process	Root hydrotropic phenotype	References
*axr1-3*	Auxin response	Increment of root bending.	Takahashi et al. (2002)
*axr2-1*	Auxin response	Increment of root bending.	Takahashi et al. (2002)
*aux1-7*	Auxin transport	Increment of root bending.	Takahashi et al. (2002)
*wav6-52*	Auxin transport	Increment of root bending.	Takahashi et al. (2002)
*aba1-1*	ABA synthesis	Reduction of root bending.	Takahashi et al. (2002)
*pyr1-1pyl1pyl2pyl4pyl5pyl8*	ABA perception	Reduction of root bending.	Antoni et al. (2002)
*abi2-1*	ABA signal transduction	Reduction of root bending.	Takahashi et al. (2002)
*hab1-1abi1-1pp2ca-1abi2-2*	ABA signal transduction	Increment of root bending.	Antoni et al. (2002)
*snrk2.2snrk2.3*	ABA signal transduction	Reduction of root bending.	Dietrich et al. (2017)
*atg2*	Autophagy	Lack of root hydrotropism.	Jimenez-Nopala et al. (2018)
*atg8b*	Autophagy	Lack of root hydrotropism.	Jimenez-Nopala et al. (2018)
*atg8i*	Autophagy	Lack of root hydrotropism.	Jimenez-Nopala et al. (2018)
*atg9*	Autophagy	Lack of root hydrotropism.	Jimenez-Nopala et al. (2018)
*bri1-5*	Brassinosteroid perception	Reduction of root bending.	Miao et al. (2018)
*eca1-3*	Calcium homeostasis	Increment of root bending.	Shkolnik et al. (2018)
*pcap1-1, pcap1-4*	Calcium signal transduction	Reduction of root bending.	Tanaka-Takeda et al. (2019)
*ipt1ipt3ipt5ipt7*	Cytokinin synthesis	Reduction of root bending.	Chang et al. (2019)
*cyp735a1*	Cytokinin synthesis	Reduction of root bending.	Chang et al. (2019)
*log2*	Cytokinin synthesis	Reduction of root bending.	Chang et al. (2019)
*ahk2-5cre1-2*	Cytokinin perception	Reduction of root bending.	Chang et al. (2019)
*ahp1ahp2ahp3*	Cytokinin signal transduction	Reduction of root bending.	Chang et al. (2019)
*arr16arr17*	Cytokinin signal transduction	Reduction of root bending.	Chang et al. (2019)
*phyAphyB*	Light perception	Reduction of root bending.	Moriwaki et al. (2012)
*hy5-1*	Light signal transduction	Reduction of root bending.	Moriwaki et al. (2012)
*wav3-1*	Protein degradation	Increment of root bending.	Takahashi et al. (2002)
*rbohC*	ROS production	Increment of root bending.	Krieger et al. (2016)
*apx1-2*	ROS scavenger	Reduction of root bending.	Krieger et al. (2016)
*pgm-1*	Starch synthesis	Increment of root bending.	Takahashi et al. (2003)
*miz2*	Vesicle trafficking	Lack of root hydrotropism.	Miyazawa et al. (2009)
*pldζ2*	Vesicle trafficking / signal transduction	Reduction of root bending.	Taniguchi et al. (2010)
*miz1*	Unknown	Lack of root hydrotropism.	Kobayashi et al. (2007)
*wav2-1*	Unknown	Increment of root bending.	Takahashi et al. (2002)
*ahr1*^a^	N.A.	Increment of root bending.	Saucedo et al. (2012)
*nhr1*^a^	N.A.	Lack of root hydrotropism.	Eapen et al. (2003)

N.A. not applicable

^a^Only the genetic locus is reported

The hydrotropic response of the legume *Lotus japonicus* is not inhibited by auxin transport inhibitors, similar to that observed in Arabidopsis. Treatment of *L. japonicus* seedlings with 3-chloro-4-hydroxyphenylacetic acid (CHPAA) or TIBA did not inhibit the hydrotropic response, but did inhibit the gravitropic response (Nakajima et al. 2017). The hydrotropic response of *L. japonicus* seedlings was not inhibited by the auxin antagonist PCIB (Nakajima et al. 2017), whereas PCIB did inhibit hydrotropism in Arabidopsis. The auxin synthesis inhibitor kynurenine blocked the development of hydrotropic root bending without preventing root growth in *L. japonicus* (Nakajima et al. 2017), whereas kynurenine did not inhibit the hydrotropic response in Arabidopsis. These combined results suggest that the role of auxin in the hydrotropic response differs between Arabidopsis and *L. japonicus*.

**Fig. 3:**
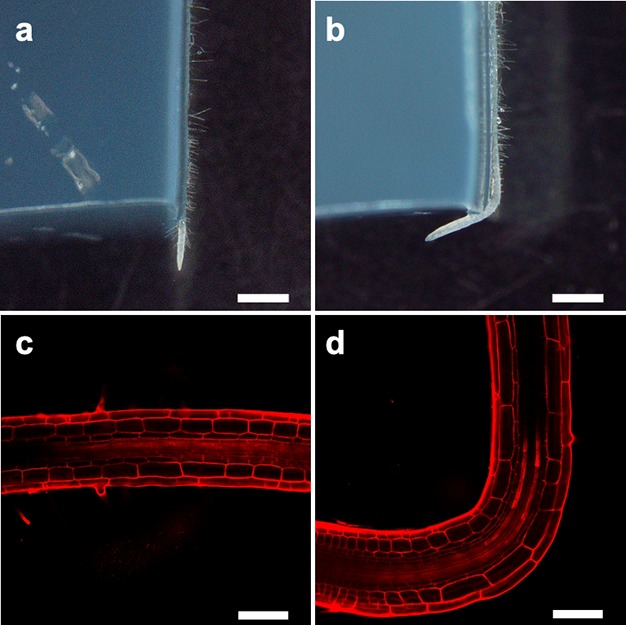
Hydrotropic response of Arabidopsis seedling root. **a** and **b** Response of Arabidopsis roots to hydrostimulation. Roots were grown in either humid (**a**) or hydrostimulated (**b**) conditions for 12 h and then photographed. **c** and **d** Confocal laser scanning micrographs of bending regions in roots. After the assay, roots grown under humid conditions (**c**) and hydrostimulated conditions (**d**) were stained with propidium iodide and imaged under a confocal laser scanning microscope. Scale bars in **a** and **b** indicate 1 mm; scale bars in **c** and **d** indicate 100 µm

Abscisic acid (ABA) has an important role in Arabidopsis root hydrotropism. Mutants defective in ABA synthesis, perception, and response displayed reduced hydrotropic responses (Antoni et al. 2013; Dietrich et al. 2017; Takahashi et al. 2002). Specific expression of the ABA signaling kinase SNF1-related protein kinase2.2 (SnRK2.2) in root cortex rescued the ahydrotropic phenotype of double mutants lacking *SnRK2.2* and *SnRK2.3*, whereas expression of *SnRK2.2* in root cap, epidermis, and endodermis did not rescue the mutant phenotype (Dietrich et al. 2017). This strongly suggests that hydrotropism is controlled by a novel growth mechanism localized in the cortex. By contrast, gravitropic growth is driven primarily by the differential expansion of epidermal cells (Swarup et al. 2005). Our observation clearly showed that root cortical cells, but not epidermal cells, differentially expanded at the bending region as a result of the root hydrotropic response (Fig. 3). Our transcriptomic analyses revealed that the degree of overlap between hydrostimulation-responsive genes and ABA-responsive genes was much greater than would be expected for randomly extracted genes (Moriwaki et al. 2010). Arabidopsis phospholipase Dζ2 (PLDζ2) was necessary for both gravitropism and hydrotropism, although the knockout mutant phenotype is quite weak (Taniguchi et al. 2010). ABA treatment increased *PLDζ2* expression, and PLDζ2 was reported to positively regulate root gravitropism by controlling auxin transport and response (Li and Xue 2007; Taniguchi et al. 2010). Several lines of evidence suggest the importance of phosphatidic acid for intercellular signaling in plants (Wang 2005). It is not yet clear how PLDζ2 activity differentiates between hydrotropism and gravitropism; it is possible that phosphatidic acid generated by PLDζ2 activity has a role in cellular response to hydrostimulation.

ABA has important roles in protective processes related to water deficit (Yamaguchi-Shonozaki and Shinozaki 2006); therefore, its involvement in hydrotropism is not unexpected. ABA enhanced pea seedling root bending that was induced by unilateral application of Ca^2+^ at the root tip, which suggests an ABA-mediated differential growth mechanism in pea (Takahashi et al. 1992). Conversely, Ponce et al. (2008) reported that ABA accelerated the ahydrotropic phenotype of the *no hydrotropic response1* (*nhr1*) mutant, and localized ABA application phenocopied the *nhr1* mutant. Treatment of wild-type seedlings with ABA also phenocopied the *nhr1* mutant (Ponce et al. 2008). Quiroz-Figueroa et al. (2010) reported that the *nhr1* ahydrotropic phenotype was suppressed by an ABA synthesis inhibitor, and the ABA content in the *nhr1* mutant was higher than that of wild type. These results suggest that ABA negatively regulates hydrotropism in Arabidopsis. However, their experimental assay system also enhanced the gravitropic response in wild-type seedlings germinated in the presence of ABA (Ponce et al. 2008), and ABA is reported to maintain root growth under osmotic stress conditions (LeNoble et al. 2004). We do not currently have a rationale that integrates both of these observations; nevertheless, it is evident that ABA has an important role (either stimulatory, inhibitory, or both depending on its endogenous concentration) in regulating root hydrotropism in Arabidopsis.

Cytokinin has also been reported to affect hydrotropic response in Arabidopsis (Saucedo et al. 2012). Exogenous application of kinetin eliminated the altered hydrotropic response phenotype of *ahr1*, while it did not affect hydrotropic response in wild type (Saucedo et al. 2012). Although the responsible gene for *ahr1* has not been discovered, *ahr1* mutant can maintain cell production at a high rate under water potential gradient, which might be the cause of robust root growth across the water potential gradient (Salazar-Blas et al. 2017). Recently, Chang et al. (2019) reported that cytokinin distributed asymmetrically during root hydrotropic response in Arabidopsis, which led to asymmetric cell production at the lower water potential side of the root. They also showed that the formation of this asymmetric distribution is MIZ1-dependent and suggested this differential cell production caused hydrotropic root bending. Exogenous application of a cytokinin, benzylaminopurine, caused greater inhibition of root growth in MIZ1-overexpressors than in the wild type, while *miz1*-*1* mutant was slightly resistant to the application of benzylaminopurine (Moriwaki et al. 2011). These results suggest intimate relationships among root hydrotropism, MIZ1 function and cytokinin, however, require further investigation especially in terms of functional relationships between MIZ1 and cytokinin.

### Signaling factors that may participate in mediating root hydrotropism

Extensive research has demonstrated that Ca^2+^ is a crucial intracellular second messenger and contributes to long-distance signaling in plants (Choi et al. 2016; Kulda et al. 2018). Gravitropism and hydrotropism in pea are both inhibited by the calcium chelator ethylene glycol-bis-(β-aminoethyl ether)-N,N,N′,N′-tetraacetic acid (EGTA) (Takahashi and Suge 1991). The calcium channel blocker lanthanum inhibited the hydrotropic response, whereas the calcium ionophore A23187 enhanced hydrotropic root bending, suggesting that apoplastic calcium influx across the plasma membrane is required for the hydrotropic response in pea (Takano et al. 1997). The hydrotropic response in Arabidopsis was reduced by EGTA but was not affected by lanthanum (Takahashi et al. 2009). Shkolnik et al. (2018) monitored cytosolic Ca^2+^ levels during the hydrotropic response in Arabidopsis, and found that a slow, long-distance, asymmetrical cytosolic Ca^2+^ signal was generated in response to hydrostimulation. This Ca^2+^ signal was transmitted through the phloem from the root cap to the elongation zone, where Ca^2+^ dispersed laterally and asymmetrically along with root bending, with higher Ca^2+^ levels in the convex tissues than in the concave tissues (Shkolnik et al. 2018). The authors identified an endoplasmic reticulum (ER)–localized calcium pump (ECA1) that was involved in this process, and MIZ1 inhibited ECA1 activity (Shkolnik et al. 2018). Although these results support the importance of root cap cells in water potential sensing, the role of the root cap in Arabidopsis root hydrotropism is still a matter of debate (Dietrich et al. 2017; Miyazawa et al. 2008). Recent work showed that the plasma membrane–associated Ca^2+^-binding protein PCaP1 was involved in Arabidopsis hydrotropism (Tanaka-Takeda et al. 2019). *PCaP1* knockout mutants displayed reduced hydrotropic root bending compared with the wild type, which was rescued by endodermis-specific expression of PCaP1. PCaP1 internalization from the Arabidopsis plasma membrane was induced by hydrostimulation but not by gravistimulation. Considering the ubiquity of Ca^2+^ signaling among diverse organisms, these results suggest that mechanisms regulating cytosolic Ca^2+^ levels and/or mechanisms decoding Ca^2+^ signals are important for the hydrotropic response. Future investigations should verify these results and determine whether these mechanisms are common in other plant species.

Reactive oxygen species (ROS) are ubiquitous signaling molecules in bacteria, animals, and plants. A recent study reported that an asymmetrical distribution of H_2_O_2_ (visualized by staining with dihydrorhodamine 123) occurred during the gravitropic response but not during the hydrotropic response in Arabidopsis (Kreiger et al. 2016). Pharmacologically or genetically mediated reduction in ROS attenuated root gravitropism and enhanced root hydrotropism (Kreiger et al. 2016). This suggests that ROS has an inhibitory role in root hydrotropism. Treatment of Arabidopsis seedlings with methyl viologen did not affect the hydrotropic response (Ponce et al. 2017), and H_2_O_2_ accumulation was observed during the hydrotropic response (Jiménez-Nopala et al. 2018). This H_2_O_2_ accumulation did not occur in the autophagy mutant *atg9*, which was defective in the hydrotropic response (Jiménez-Nopala et al. 2018). Autophagy was involved in hydrostimulation-induced amyloplast degradation in Arabidopsis root (Nakayama et al. 2012). Amyloplast degradation also was observed in radish (*Raphanus sativus* L.) hydrotropism, but not in pea and cucumber hydrotropism (Takahashi et al. 2003, 2009). Thus, H_2_O_2_ and/or autophagy may be involved in the hydrotropic response, at least in a species-specific manner. Further studies are needed to fully elucidate the roles of ROS and autophagy in plant hydrotropism.

### Genetic regulation of Arabidopsis root hydrotropism

Genetic screens to isolate mutants with defective hydrotropism have identified four mutants in Arabidopsis. These include three ahydrotropic mutants (*nhr1*, *miz1*-*1*, and *miz2*), and the *altered hydrotropic response1* (*ahr1*) mutant, which develops an extensive root system oriented toward a high water potential area in the presence of a water potential gradient (Eapen et al. 2003; Kobayashi et al. 2007; Miyazawa et al. 2009; Saucedo et al. 2012). Although the responsible genes for *nhr1* and *ahr1* have not been reported, genetic and phenotypic analyses revealed that (1) both *nhr1* and *ahr1* are semi-dominant mutants, (2) application of ABA or cytokinin altered *nhr1* and *ahr1* phenotypes, (3) *nhr1* and *ahr1* mutants retain starch granules under hydrostimulated conditions, and (4) the proliferative activity of *ahr1* root apical meristem was maintained under a water potential gradient (Cassab et al. 2013; Salazar-Blas et al. 2017). To further identify the precise roles of *NHR1* and *AHR1*, and the physiological significance of these mutations on root hydrotropism, it will be crucial to identify the responsible genes.

The genes responsible for the *miz1*-*1* and *miz2* mutants have been identified. Neither of these mutants display root hydrotropism, although they have normal root gravitropism. Although the phenotypes of both mutants are similar, the functions of the encoded proteins appear to differ. *MIZ1* encodes a protein that contains a domain of unknown function (DUF617), which we designate as the MIZ1-domain (Kobayashi et al. 2007). *MIZ2* encodes a guanine-exchange factor for ADP-ribosylation factor–type G proteins (ARF-GEF; Miyazawa et al. 2009).

Expression analyses using green fluorescent protein (GFP)–fused MIZ1 (MIZ1-GFP) indicate that MIZ1-GFP is expressed specifically in the epidermis, cortex, lateral root cap, and columella cells (Yamazaki et al. 2012). Biochemical analyses indicate that MIZ1-GFP is a soluble protein associated in part with the endoplasmic reticulum (Yamazaki et al. 2012). *MIZ1* expression is enhanced by ABA treatment, blue light irradiation, and abiotic stresses such as osmotic, cold, and drought stresses (Miyazawa et al. 2012; Moriwaki et al. 2012). However, MIZ1-GFP was not differentially localized in convex and concave tissues during the hydrotropic response (Moriwaki et al. 2013). Recent analyses show that cortex-specific expression of MIZ1-GFP is sufficient to rescue the ahydrotropic phenotype of *miz1*-*1* (Dietrich et al. 2017). Currently, we do not know the functional role of MIZ1 protein in tissues other than cortex. A recent study reported that MIZ1 directly binds to the ECA1 Ca^2+^ pump and negatively regulates its activity, and hydrostimulation elevates the cytoplasmic Ca^2+^ level in wild-type columella cells, which is diminished in the *miz1* mutant (Shkolnik et al. 2018). The auxin content and lateral root initiation in *MIZ1*-overexpressing plants were reduced compared with those of wild-type plants (Moriwaki et al. 2011). Plant roots preferentially develop lateral roots toward the direction of higher water availability, which involves local auxin synthesis and transport (Bao et al. 2014). Thus, MIZ1 may function in regulating root system architecture by controlling auxin synthesis and/or metabolism when roots are exposed to a water potential gradient.

To our knowledge, the MIZ1-domain is encoded specifically in land plants, suggesting that the emergence of this domain is related to the evolution of land plant species. In addition to MIZ1, there are another 11 MIZ1-domain–encoding genes (*MIZ1*-*LIKE* genes, or *MIL*s) in the Arabidopsis genome, and other flowering plants also encode more than ten *MIL*s in their genome. The moss *Physcomitrella patens* expresses three MIZ1-domain–encoding genes, although this species does not have roots as a water-absorbing organ (Fujii et al. 2018; Kobayashi et al. 2007). This suggests that the original function of the MIZ1-domain is not necessarily related to hydrotropism. Recent phylogenic analyses showed that not all angiosperms express *MIZ1* orthologs (Fujii et al. 2018). Putative *MIZ1* orthologs have been identified in dicots (cucumber, soybean, and tomato) but not in monocots (rice and maize). It remains to be determined whether the putative MIZ1 ortholog functions in cucumber hydrotropism, which has a different hydrotropic mechanism than Arabidopsis. Analyses of other *MIL*s will be crucial to determine the MIZ1-domain function.

Identification of the *MIZ2* gene responsible for the *miz2* mutant revealed an indispensable role for membrane trafficking in hydrotropism. *MIZ2* encodes GNOM, an ARF-GEF that is required for vesicle formation. GNOM mediates polar recycling of PIN proteins, which are involved in auxin efflux (Singh and Jürgens 2018). Treatment with the fungal toxin brefeldin A, which inhibits GNOM activity, also abolishes the hydrotropic response in wild-type roots (Miyazawa et al. 2009). However, PIN1 and PIN2 localization in the *miz2* mutant does not differ from that in the wild type (Miyazawa et al. 2009; Moriwaki et al. 2012), suggesting that GNOM differentially regulates hydrotropism and PIN localization. Rather, GNOM may regulate the localization of other membrane proteins that are indispensable for root hydrotropism. Several membrane proteins have been proposed as hydraulic sensor candidates, including mechanosensitive channels, wall-associated kinases, receptor-like kinases, and Arabidopsis Histidine Kinase1 (Christmann et al. 2013). These proteins monitor membrane tension, cell wall tension and distortion, and turgor pressure, respectively. It remains to be determined whether GNOM regulates the localization of these proteins.

The *miz2* mutation suppressed all pleiotropic effects caused by *MIZ1* overexpression, such as slow root elongation, lateral root development retardation, and enhanced root hydrotropism (Miyazawa et al. 2012; Moriwaki et al. 2011). This indicates that GNOM acts upstream of MIZ1 and has a functional relationship with MIZ1, either directly or indirectly. Our data indicate that the subcellular localization of MIZ1 and GNOM differs. It remains to be determined how MIZ1 and GNOM interact to clarify the molecular mechanism mediating the hydrotropic response in Arabidopsis.

## Perspectives

The ecological significance of root hydrotropism has been questioned by some researchers, especially as different studies report inconsistent results (e.g., Cole and Mahall 2006; Tsuda et al. 2003). We have shown that different plant species have different mechanisms mediating root hydrotropism, and the relationship between hydrotropism and gravitropism differ among different species. Thus, it is necessary to select appropriate experimental models and establish standardized experimental set ups to clarify the ecological significance of root hydrotropism. Studies using Arabidopsis showed that lateral roots respond to water potential gradients and display positive hydrotropism in a MIZ1-dependent manner (Iwata et al. 2012). An experimental system was established to study Arabidopsis hydrotropism in soil, and the shoot biomass and survival rate of wild-type, *miz1* mutant, and *MIZ1*-overexpressing plants were examined (Iwata et al. 2013). The results clearly showed that wild-type plants respond to moisture gradients in soil and grew many roots toward the moistened area, which was pronounced in roots of *MIZ1*-overexpressing plants. By contrast, *miz1* mutant roots did not respond to the moisture gradient in soil and grew vertically. The shoot biomass and the number of plants that survived under water-limited conditions were much greater in *MIZ1*-overexpressing plants than those in the wild type and *miz1* mutant. These results clearly indicate that root hydrotropism contributes to root system architecture, plant productivity and survival under water-limited conditions. A recent report by Miao et al. (2018) showed that the strength of the hydrotropic response varies among Arabidopsis ecotypes and correlates with their geographic distribution. Ecotypes that originate in relatively dry regions display strong hydrotropic responses, whereas ecotypes that originate in relatively humid regions display weak hydrotropic responses (Miao et al. 2018).

**Fig. 4:**
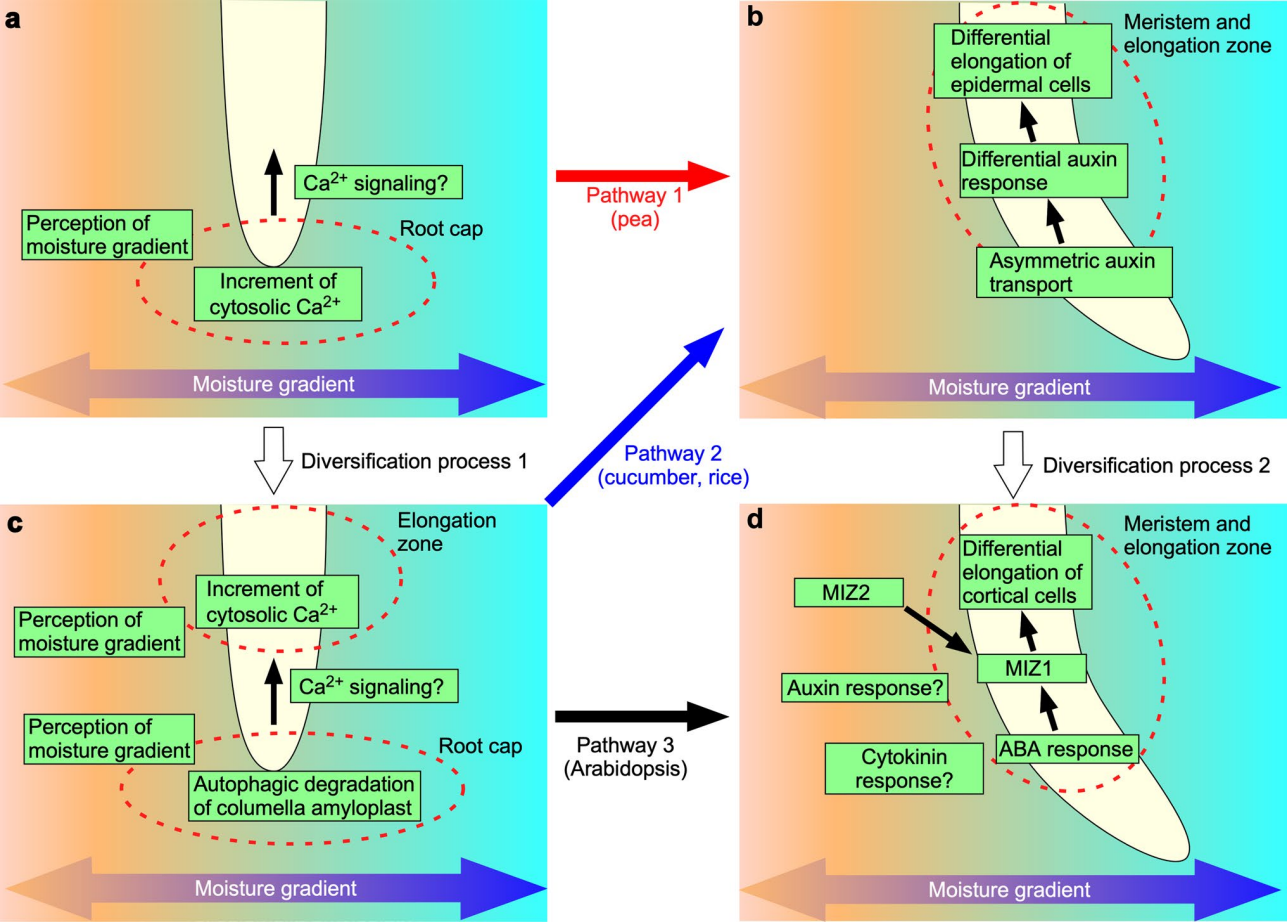
Our current hypothesis on diversification of the mechanism of hydrotropism. **a** Sensing mechanism used in pea. **b** Sensing mechanism used in Arabidopsis, cucumber and rice. **c** Tropic growth mechanism used in pea, cucumber and rice. **d** Tropic growth mechanism used in Arabidopsis. Species specific pathways are depicted by arrows in red, blue and black. Two proposed diversification processes are also in white arrows. See text for details

Eapen et al. (2017) examined the correlation between the hydrotropic response strength and plant growth under drought conditions in a field experiment using maize hybrid lines. They selected three robust hydrotropic responders and three weak hydrotropic responders for the field study. The results showed that two robust hydrotropic responders had significantly higher root weight than other tested plants, and the grain yield of one of the robust hydrotropic responders was higher than that of the others under partial lateral irrigation conditions (Eapen et al. 2017). Root weight and grain yield were positively correlated in robust hydrotropic responders, whereas root weight and grain yield were not positively correlated in weak hydrotropic responders subjected to drought, partial lateral irrigation, and normal irrigation conditions (Eapen et al. 2017). These results directly indicate the ecological significance of root hydrotropism, and similar trials combined with genetic analyses will facilitate the development of new drought-resistant crop cultivars. Several plant species have displayed root hydrotropic responses, including beneficial crops such as maize, wheat, rice, pea, and cucumber (Mizuno et al. 2002; Nakajima et al. 2017: Oyanagi et al. 1995; Takahashi and Scott 1991, 1993). So far, we do not know why the molecular mechanisms of root hydrotropism are so different. Because the molecular mechanism of root hydrotropism in two legume species, Pea and *L. japonicus*, differed, we assume that the acquisition of capability of root hydrotropism occurred during the diversification of Fabaceae family. Our current hypothesis for the diversification of mechanism of hydrotropism is illustrated in Fig. 4. Originally, root hydrotropism was driven by a mechanism that resembles to that of gravitropism (Fig. 4, pathway 1). During speciation, the elongation zone of some plant species acquired the ability to express a set of genes which are required for sensing moisture gradients (Fig. 4, diversification process 1). Then, some species began to use ABA-mediated growth mechanism instead of auxin-mediated tropic growth (Fig. 4, diversification process 2). These processes led to the birth of new pathways (Fig. 4, pathways 2 and 3). To understand why and how the mechanisms of hydrotropism were diversified, comparative analyses on the mechanisms of root hydrotropism using wild species and on the environmental factors of the places where they speciated would be important.

Plant roots position new lateral root branches according to the water availability across the circumferential axis of the root (Bao et al. 2014). Lateral root gravitropism in Arabidopsis was enhanced by water deficit, and the roots grew deeper into the soil (Rellán-Álvarez et al. 2015). These responses have the potential to contribute to drought avoidance under field conditions. These two phenomena depend on auxin signaling, which is critical for gravitropism, but not for hydrotropism, in Arabidopsis roots. It is possible that acquisition of these responses could be a factor that accelerates modification of an auxin-dependent mechanism of root hydrotropism into an auxin-independent one. Although the experiments require careful design and execution to separate hydrotropic and gravitropic responses, it will be informative to determine how plant roots differentially respond after sensing a water potential gradient.
