# Correction to: Molecular mechanisms mediating root hydrotropism: what we have observed since the rediscovery of hydrotropism

**DOI:** 10.1007/s10265-020-01179-y

**Published:** 2020-03-25

**Authors:** Yutaka Miyazawa, Hideyuki Takahashi

**Affiliations:** 1 Faculty of Science, Yamagata University, 1-4-12 Kojirakawa-machi, Yamagata, 990-8560 Japan; 2 Graduate School of Life Sciences, Tohoku University, 2-1-1 Katahira, Aoba-ku, Sendai, 980-8577 Japan

## Correction to: Journal of Plant Research (2020) 133:3–14 https://doi.org/10.1007/s10265-019-01153-3

The article Molecular mechanisms mediating root hydrotropism: what we have observed since the rediscovery of hydrotropism, written by Yutaka Miyazawa and Hideyuki Takahashi, was originally published Online First without Open Access. After publication in volume 133, issue 1, pages 3–14 the author decided to opt for Open Choice and to make the article an Open Access publication. Therefore, the copyright of the article has been changed to © The Author(s) 2020 and the article is forthwith distributed under the terms of the Creative Commons Attribution 4.0 International License (https://creativecommons.org/licenses/by/4.0/), which permits use, duplication, adaptation, distribution and reproduction in any medium or format, as long as you give appropriate credit to the original author(s) and the source, provide a link to the Creative Commons license, and indicate if changes were made.

The original article was updated.

